# Association between dietary patterns and obesity-related metabolic phenotypes in Chinese middle-aged and older adults: a cross-sectional study

**DOI:** 10.1038/s41598-025-18446-4

**Published:** 2025-10-06

**Authors:** Fangfang Pu, Jialing Lin, Rui He, Yaoyao Wei, Jingjing Li, Xinyi Liao, Jiuming Yan, Yan Wang, Lei Shi, Xianchun Zeng, Wen Hu

**Affiliations:** 1 Department of Clinical Nutrition, West China Hospital, Sichuan University, 37 Guoxue Alley, Wuhou District, Chengdu, 610041 Sichuan China; 2 Laboratory Medicine Center, Sichuan Tianfu New Area People’s Hospital, No. 97 Zhengbei Shangjie, Huayang Street, Tianfu New Area, Chengdu, 610213 Sichuan China; 3 Department of Nutrition and Food Hygiene, West China School of Public Health and West China Fourth Hospital, Sichuan University, Chengdu, 610041 Sichuan China; 4 School of Laboratory Medicine, Chengdu Medical College, No. 783, Xindu Avenue, Xindu District, Chengdu, 610500 Sichuan China

**Keywords:** Middle-aged and older adults, Dietary patterns, Obesity metabolic phenotype, Metabolic abnormality, Diseases, Endocrinology, Risk factors

## Abstract

Middle-aged and elderly people are prone to obesity or metabolic abnormalities, and an unreasonable dietary pattern is an important factor affecting the occurrence or development of metabolic diseases. 15,160 middle-aged and elderly participants classified into four categories on the basis of obesity metabolic phenotype criteria: metabolically healthy nonobese (MHNO), metabolically unhealthy nonobese (MUNO), metabolically healthy obese (MHO), and metabolically unhealthy obese (MUO). The main dietary patterns of the study population were identified via food frequency questionnaire and principal component analysis. A multi-categorical logistic regression model was used to observe the relationships between dietary patterns and different obesity metabolic phenotypes in middle-aged and elderly people. A total of four dietary patterns were extracted. Higher scores for the “egg-dairy preference” pattern were associated with a reduced risk of MUNO, MHO, and MUO. A high consumption of the “plant preference” pattern was also associated with a reduced risk of MUNO. Conversely, a high intake of the “grain and meat preference” pattern was associated with the highest prevalence of MHO. Our study revealed a strong association between a diet rich in eggs and dairy and a lower prevalence of the obesity-related metabolic phenotype in middle-aged and elderly people.

## Introduction

 According to the “World Social Reports 2023” survey, it is projected that by 2050, the global elderly population will double, and China will become the most aged country in Asia^1^. Prolonged work duration, mental stress, unhealthy diets and age-related physiological changes can predispose middle-aged and elderly individuals to obesity or metabolic disorders^2,3^. It is worth noting that compared with obesity alone or metabolic abnormalities alone, obesity accompanied by metabolic abnormalities will significantly increase the risk of cardiovascular disease, cerebrovascular disease, hyperlipidemia, diabetes, hyperuricemia and other diseases^4–8,9,10^, thus adversely affecting health. Hence, early detection of obesity and metabolic abnormalities is imperative for disease prevention.

Obesity metabolic phenotypes are usually categorized into four phenotypes on the basis of body mass index (BMI) and metabolic status as follows: metabolically healthy nonobese (MHNO), metabolically unhealthy nonobese (MUNO), metabolically healthy obese (MHO), and metabolically unhealthy obese (MUO). The emergence and progression of these phenotypes are often intertwined with genetic predispositions, geographical factors, and dietary habits, among other factors^11–13^. Research indicates that these phenotypes can evolve over time on the basis of an individual’s physiological condition, with MHO representing a transitional state in the spectrum of metabolic disorders^14,15^. Longitudinal studies, such as the one conducted by Soriguer et al.^16^, have revealed important insights into the dynamics of these phenotypes. Over a 6-year follow-up period, a significant portion of individuals initially classified as MHO exhibited metabolic abnormalities and faced an elevated risk of developing type 2 diabetes, akin to MUO subjects^17^. Moreover, findings from a 2.2-year follow-up indicated that while a majority of individuals categorized as MHNO and MUO at baseline remained within their respective states, only approximately half of those classified as MHO and MUNO remained unchanged, with a considerable proportion of MHO individuals transitioning to MUO^18^. These observations underscore the critical importance of accurately assessing obesity metabolic phenotypes and comprehensively analyzing the associated factors influencing their development and progression.

Numerous studies have highlighted the association between diet and prevalent chronic diseases in specific regions, emphasizing the importance of adopting rational dietary patterns and healthy lifestyles^19,20^. Dietary patterns offer a holistic approach to understanding how the combined effects of foods and nutrients influence chronic disease development. Several clinical investigations have demonstrated the efficacy of caloric restriction in improving body composition, mitigating inflammation and oxidative stress, and regulating glucose and lipid metabolism, thereby reversing conditions such as obesity, type 2 diabetes, and atherosclerosis^21–23^. Recent research has indicated that adherence to a plant-based diet is linked to a reduced risk of all-cause mortality and cardiovascular-related mortality. Moreover, these findings suggest that the sooner individuals modify their dietary habits, the greater the metabolic benefits they may experience^24^. Given China’s vast geographical expansion and diverse climatic conditions, notable regional variations in dietary habits and characteristics exist^25^. Regional dietary pattern studies have shown that residents’ dietary preferences are influenced by specific environmental factors, aiding in tailoring more effective dietary interventions^26,27^. Sichuan, renowned for its distinctive food culture dating back to the Han Dynasty, has a penchant for spicy cuisine^28^. People in Sichuan prefer sausages, bacon, Sichuan pickles, spicy flavors, and cooking methods with many oils, salts and spices. However, with economic development, dietary patterns have evolved, exhibiting variations across different age groups, genders, and levels of education^29,30^. Gathering evidence from diverse regions of China, each characterized by unique dietary patterns, is crucial for preventing various metabolic phenotypes of obesity and tailoring interventions accordingly.

Through a comprehensive analysis of the dietary habits of middle-aged and elderly people in Sichuan Province and a study of the relationships between their dietary patterns and different obesity metabolic phenotypes, we sought to identify the key dietary factors influencing the different metabolic states of this population. This understanding will enable the development of targeted nutritional interventions that address specific dietary needs and promote healthier lifestyle choices.

## Methods and materials

### Participants

This cross-sectional study was nested within the “Natural Population Health Cohort Study of West China Hospital of Sichuan University”, which is a large prospective cohort study focused on the health status of residents living in Southwest China. Convenience sampling was employed to recruit participants aged 45 years and older during health examinations at three cohort sites in Mianzhu, Longquan, and Pidu districts of Sichuan Province. The exclusion criteria included individuals with serious or terminal illnesses, those unable to communicate normally, and those who could not cooperate with the questionnaire, laboratory tests, or other required assessments. Ethical approval was given by the Biomedical Ethics Review Committee of West China Hospital of Sichuan University with reference number NO. 2021–752, and participants gave written informed consent prior to participation in the study. All methods were performed in accordance with the relevant guidelines.

**Fig. 1:**
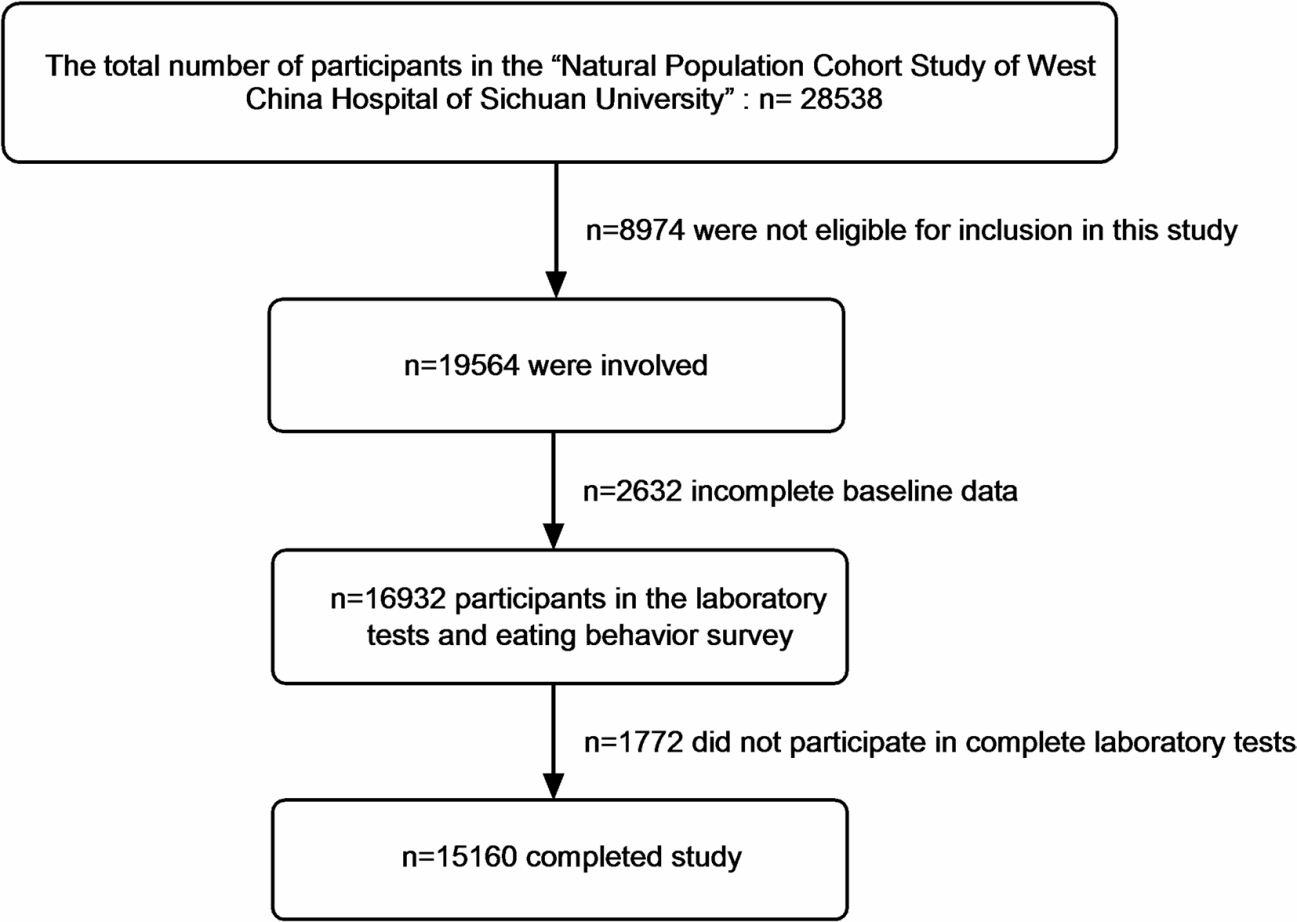
The flowchart for the screening of research subjects.

This study was conducted between June 2019 and August 2021. A total of 15,160 participants were included for further analysis, with the participant screening flowchart detailed in Fig. 1.

### Questionnaire surveys

The baseline questionnaire was administered via one-on-one interviews conducted by uniformly trained investigators. This questionnaire collected detailed information on participants’ demographic characteristics (age, gender, marital status, education level, and employment status), lifestyle habits (smoking, drinking, exercise, and sleep), mental health indicators (anxiety and depression), and family history of diseases, including diabetes mellitus, hypertension, and hyperlipidemia. Lifestyle habits and mental health indicators were evaluated in accordance with our previous study^31^. with operational definitions as follows: ①Smoking: current smoking behavior (≥ 4 cigarettes per week in the past six months). ②Alcohol consumption: regular drinking (≥ 1 occasion per week in the past six months). ③Sleep problems: assessed via the Pittsburgh Sleep Quality Index, with scores > 10 indicating poor sleep. ④Anxiety and depression: measured using the Generalized Anxiety Disorder-7 and Patient Health Questionnaire-9 scales, respectively.

### Dietary survey and identification of dietary patterns

Dietary intake was evaluated using a simplified food frequency questionnaire (SFFQ) pre-validated for the local population. In 145 Chengdu community residents, the SFFQ’s reliability (4-week test-retest) and validity (against 3-day 24-h dietary recall) were assessed, showing acceptable outcomes: test-retest coefficients ranged 0.380–0.637 for energy/nutrients and 0.318–0.703 for foods, while validity correlations were 0.412–0.771 (energy/nutrients) and 0.186–0.883 (foods). The questionnaire, comprising 12 major food groups with five frequency response categories (ranging from “almost never” to “one or more times per day”), follows design specifications consistent with the methodology reported in Wei Yaoyao et al.’s study^32^. To ensure quantitative precision in dietary assessment, a range of food portion models and standardized containers were employed as measurement criteria. For each food item in the SFFQ, a corresponding portion model was developed, with each model representing a defined serving size and assigned a specific gram weight. Following standardization of consumption data across all 12 food groups, factor analysis was performed to identify predominant dietary patterns.Varimax rotation was applied to improve interpretability. Four factors were extracted after the evaluation of the eigenvalues (greater than 1.0) and the scree test. Food groups with a factor loading greater than |0.40| were considered the main contributors to each dietary pattern and were representative of its characteristics. The factors were subsequently named descriptively on the basis of the predominant food groups associated with each respective pattern as follows: “plant preference” pattern, “grain-meat preference” pattern, “meat preference” pattern, and “egg-dairy preference” pattern.

### Assessment of the obesity metabolic phenotypes

Height and weight were measured via an electronic height and weight meter (single measurement). The participants were instructed to stand barefoot, wear light clothing, and fast overnight before the measurement. Blood pressure (BP) was measured from the upper right arm via an upper-arm sphygmomanometer after 5 min of rest in a seated position (Measure three times and take the average). Blood samples for the analysis of fasting blood glucose (FBG) and lipids were collected in siliconized vacuum plastic tubes and analyzed via a Beckman Coulter Chemistry Analyzer (AU5800).

The metabolic obesity phenotypes were categorized into four groups on the basis of the presence of obesity and metabolic abnormalities as follows: MHNO, MUNO, MHO, and MUO. Obesity was defined as a body mass index(BMI) ≥ 28 kg/m² according to the adult BMI standard recommended by the Chinese Expert Consensus on Medical Nutritional Treatment of Overweight/Obesity (2016 edition)^33^. Metabolic abnormalities were defined as meeting two or more of the following criteria: (1) TG ≥ 1.7 mmol/L or the use of lipid-lowering drugs; (2) HDL-C < 1.04 mmol/L for men or < 1.29 mmol/L for women; (3) FBG ≥ 5.6 mmol/L or the use of diabetes medications; and (4) systolic blood pressure ≥ 130 mmHg or diastolic blood pressure ≥ 85 mmHg or the use of antihypertensive drugs^31^.

### Quality control

Before initiating the survey, the investigators underwent standardized training and assessment conducted by the research team to ensure uniformity. The language used during the survey was standardized to maintain consistency in the measurements. The instruments were subjected to rigorous quality control and calibration prior to data collection. Dietary intake was quantified via a standardized food model. The data collected were verified and entered by two independent individuals, with both manual and computer-assisted logic error detection employed to increase accuracy.

### Statistical analysis

Continuous variables are presented as the means ± standard deviations, whereas categorical variables are reported as percentages. Intergroup differences were analyzed via one-way ANOVA combined with the least significant difference (LSD) post hoc test or the chi-square test, depending on the metabolic obesity phenotype. Scores for each dietary pattern were calculated using standardized food consumption data and categorized into four quartiles based on ascending ranks: Q1 (0–25%), Q2 (25–50%), Q3 (50–75%), and Q4 (75–100%). A multi-categorical logistic regression model was used to investigate the correlations between quartiles of dietary patterns and metabolic obesity phenotypes, as well as the associations between dietary patterns and these phenotypes. The models were adjusted for gender, age, educational level, employment status, smoking status, drinking, exercise, sleep, anxiety, depression and family history of disease. Additionally, analyses stratified by gender and age were conducted. All analyses were performed via SPSS version 26.0 software for Windows (SPSS Inc., Chicago, IL, USA). Differences were considered statistically significant at *P* < 0.05.

## Results

### Characteristics of the participants

The characteristics of the participants according to obesity metabolic phenotypes are shown in Table 1. The final enrollment in this study included 15,160 individuals, with 30.8% being male and 69.2% being female. Among these participants, 65% were aged between 45 and 60 years, whereas 35% were 60 years or older. According to the obesity metabolic phenotype classification, 55% of the participants exhibited MHNO characteristics, 31.6% were classified as MUNO, 5% were identified as MHO, and 8.4% fell into the category of MUO. Comparisons of baseline characteristics across different obesity metabolic phenotypes revealed significant differences in sex, age, BMI, education level, employment status, family history of diabetes and hypertension, weekly exercise duration, smoking, drinking, and anxiety levels (all *P* < 0.05).

**Table 1:** Participant characteristics by different obesity metabolic phenotypes/(%)or $\bar {X} \pm S$.

Characteristics	Nonobesity and metabolic normality (MHNO, *n* = 8340)	Nonobese and metabolic abnormality (MUNO, *n* = 4789)	Obesity and metabolic normality (MHO, *n* = 763)	Obesity and metabolic abnormality (MUO, *n* = 1268)	*P* value
Gender (%)					<0.001^#^
Male	28.10	34.50	26.90	36.30	
Female	71.90	65.50	73.10	63.70	
Age (years)	56.31 ± 6.92	58.79 ± 6.81	57.07 ± 6.88	58.31 ± 7.03	<0.001^*^
Body mass index (BMI, kg/m^2^)	23.35 ± 2.36^(a, b,c)^	24.38 ± 2.13^(b, c)^	29.74 ± 1.78^(c)^	30.14 ± 3.63	<0.001^*^
Education level (%)					<0.001^#^
Elementary school and below	35.30	40.30	50.60	47.20	
Secondary school and Vocational high school	59.50	55.70	46.00	48.40	
College and above	5.20	4.10	3.40	4.30	
Marital status (%)					0.970^#^
Married	92.10	92.20	92.00	92.50	
Others	7.90	7.80	8.00	7.50	
Employment status (%)					<0.001^#^
Employment	23.60	18.00	22.10	17.80	
Others	76.40	82.00	77.90	82.20	
Time of exercise per week (minutes)	262.41 ± 279.37^(a)^	286.22 ± 291.31^(b, c)^	262.54 ± 308.43	266.23 ± 276.34	<0.001^*^
Smoking (%)					<0.001^#^
Yes	14.40	17.30	10.90	15.70	
No	85.60	82.70	89.10	84.30	
Drinking (%)					<0.001^#^
Yes	22.20	26.60	22.40	27.80	
No	77.80	73.40	77.60	72.20	
Sleep problem (%)					0.255^#^
Yes	20.0	21.10	18.30	20.30	
No	80.0	78.90	81.70	79.70	
Anxiety (%)					0.001^#^
Yes	8.60	7.20	10.0	6.70	
No	91.40	92.80	90.0	93.30	
Depression (%)					0.851^#^
Yes	5.50	5.40	6.20	5.70	
No	94.50	94.60	93.80	94.30	
Family history of diseases (%)					
Diabetes	8.20	11.00	7.70	10.50	<0.001^#^
Hypertension	18.20	19.90	16.60	20.30	0.020^#^
Hyperlipidemia	1.20	1.30	0.80	1.00	0.581^#^

a vs. the MUNO group, *P* < 0.05; b vs. the MHO group, *P* < 0.05; c vs. the MUO group, *P* < 0.05; ^*^ represents ANOVA, ^#^ represents chi-square test.

### Dietary patterns

Four distinct dietary patterns were identified through factor analysis, and the factor loadings for each food group corresponding to these patterns are presented in Table 2. Together, these patterns explained 45.4% of the variance in total food intake, with the “plant preference” pattern accounting for 16.5%, the “grain-meat preference” pattern accounting for 11.9%, the “meat preference” pattern accounting for 8.6%, and the “egg-dairy preference” pattern accounting for 8.4%. On the basis of their contributions to the total variation, the dietary patterns were characterized as follows: the “plant preference” pattern, featuring high consumption of mixed grains, potatoes, fresh vegetables, fruits, legumes, and nuts; the “grain-meat preference” pattern, marked by elevated intake of rice and wheat-based refined carbohydrates, cured and smoked products and animal meat; the “meat preference” pattern, noted for high consumption of aquatic products, animal meat and offspring; and the “egg‒dairy preference” pattern, characterized by increased intake of eggs and dairy products.

The proportions of different dietary patterns in the MHNO, MUNO, MHO, and MUO methods were as follows: the “plant preference” pattern (55.1%, 30.7%, 5.2%, and 9.0%); the “grain-meat preference” pattern (52.7%, 32.7%, 4.9%, and 9.6%); the “meat preference” pattern (54.2%, 31.2%, 5.4%, and 9.3%); and the “egg-dairy preference” pattern (57.5%, 31.6%, 4.8%, and 6.1%). The distribution of each dietary pattern quartile across the different metabolic phenotypes of obesity is shown in Table 3.

**Table 2:** Factor loadings in food groups associated with dietary patterns.

Food groups	Plant preference pattern	Grain-meat preferenc pattern	Meat preference pattern	Egg-dairy preference pattern
Coarse grains and potatoes	**0.684**	−0.059	−0.223	0.037
Fruits and vegetables	**0.550**	0.073	0.122	0.087
Legumes and legume products	**0.580**	0.053	0.273	0.079
Nuts	**0.477**	0.035	0.173	0.089
Rice and flour-based	0.075	**0.717**	−0.109	−0.157
Cured and processed meat products	−0.139	**0.567**	0.218	0.257
Meat	0.107	**0.495**	**0.488**	0.054
Aquatic products	0.251	−0.124	**0.667**	0.019
Animal offal	0.020	0.102	**0.609**	−0.098
Dairy products	0.103	−0.221	0.043	**0.722**
Eggs	0.163	0.156	−0.138	**0.714**
Salted vegetables	0.327	0.395	−0.024	−0.209

Absolute values of factor loadings > 0.4 are shown in bold.

**Table 3:** Differences in the distribution of obesity-related metabolic phenotypes among the four dietary patterns (%).

Dietary patterns	Different obesity metabolic phenotypes				X^2^	*P* value
	MHNO(*n* = 8340)	MUNO(*n* = 4789)	MHO(*n* = 763)	MUO(*n* = 1268)		
Plant preference pattern					5.852	0.755
Q1	25.0	24.6	26.2	25.6		
Q2	25.3	24.8	25.0	23.6		
Q3	25.2	24.6	23.7	25.7		
Q4	24.5	25.9	25.0	25.1		
Grain-meat preference pattern					23.350	0.005
Q1	24.2	25.0	28.0	28.6		
Q2	24.8	25.8	25.2	22.8		
Q3	25.0	25.0	24.4	25.0		
Q4	25.9	24.1	22.4	23.6		
Meat preference pattern					22.646	0.007
Q1	25.2	24.5	24.8	25.8		
Q2	25.9	23.8	24.8	23.7		
Q3	25.1	25.5	24.5	22.8		
Q4	23.8	26.3	26.0	27.7		
Egg-dairy preference pattern					118.417	<0.001
Q1	26.5	24.5	21.6	19.2		
Q2	26.2	24.0	23.9	21.6		
Q3	25.1	24.9	24.8	25.2		
Q4	22.3	26.6	29.8	34.0		

### Association between dietary patterns and metabolic phenotypes of obesity

Multi-categorical logistic regression analyses were conducted to examine the associations between different dietary patterns and obesity-related metabolic phenotypes, controlling for influencing factors. The results are presented in Table 4. Compared with the “plant preference” pattern, the “meat preference” pattern was identified as a risk factor for MUNO (OR = 1.121, 95% CI: 1.004–1.252) after adjustment. Conversely, the “egg-dairy preference” pattern emerged as a protective factor for MUO (OR = 0.644, 95% CI: 0.540–0.768) after adjustment.

The quartiles of each dietary pattern are presented in Table 5. Higher scores for the “egg‒dairy preference” pattern were associated with a lower prevalence of MUNO, MHO and MUO than with the MHNO pattern. The OR for the extreme quartile was 0.728 (95% CI, 0.656–0.808) for MUNO, 0.622 (95% CI, 0.502–0.770) for MHO, and 0.465 (95% CI, 0.392–0.553) for MUO after adjustment. A high consumption of the “plant preference” pattern was associated with the lowest prevalence of MUNO. The ORs across quartiles of the “plant preference” pattern were 1 (reference), 0.933 (95% CI, 0.842–1.034), 0.934 (95% CI, 0.842–1.036), and 0.889 (95% CI, 0.801–0.988) after adjustment. Additionally, a high intake of the “grain-meat preference” pattern was associated with the highest prevalence of MHO. The ORs across quartiles of the “grain–meat preference” pattern were 1 (reference), 1.092 (95% CI, 0.877–1.358), 1.122 (95% CI, 0.900–1.399), and 1.299 (95% CI, 1.036–1.629) after adjustment.

**Table 4:** Association between four dietary patterns and metabolic phenotypes of obesity.

Dietary patterns	MHNO	MUNO		MHO		MUO	
		OR (95%CI)	*P* value	OR (95%CI)	*P* value	OR (95%CI)	*P* value
Plant preference pattern	-	.	.	.	.	.	.
Grain-meat preference pattern		1.097 (0.984–1.223)	0.096	0.920 (0.736–1.151)	0.465	1.022 (0.861–1.214)	0.803
Meat preference pattern		1.121 (1.004–1.252)	0.043	1.040 (0.833–1.298)	0.731	1.094 (0.919–1.303)	0.311
Egg-dairy preference pattern		0.996 (0.899–1.103)	0.939	0.841 (0.681–1.039)	0.108	0.644 (0.540–0.768)	<0.001

Models adjusted for gender, age, education level, marital status, employment, family history of diseases, exercise, smoking, drinking, sleep, anxiety and depression.

**Table 5:** Association between quartiles of dietary patterns and metabolic phenotypes of obesity.

Dietary patterns	MHNO	MUNO		MHO		MUO	
		OR (95%CI)	*P* value	OR (95%CI)	*P* value	OR (95%CI)	*P* value
Plant preference pattern							
Q1	-	.	.	.	.	.	.
Q2		0.933 (0.842–1.034)	0.185	0.969 (0.783-1.200)	0.775	1.041 (0.880–1.232)	0.636
Q3		0.934 (0.842–1.036)	0.195	1.078 (0.871–1.333)	0.491	0.983 (0.827–1.169)	0.850
Q4		0.889 (0.801–0.988)	0.028	1.165 (0.942–1.442)	0.159	1.049 (0.883–1.246)	0.588
Grain-meat preference pattern							
Q1	-	.	.	.	.	.	.
Q2		1.032 (0.930–1.144)	0.555	1.092 (0.877–1.358)	0.431	1.016 (0.855–1.207)	0.858
Q3		1.037 (0.934–1.152)	0.498	1.122 (0.900-1.399)	0.305	0.874 (0.731–1.045)	0.140
Q4		0.983 (0.879–1.099)	0.764	1.299 (1.036–1.629)	0.023	1.049 (0.876–1.257)	0.603
Meat preference pattern							
Q1							
Q2		1.004 (0.905–1.114)	0.938	1.018 (0.821–1.262)	0.870	0.869 (0.731–1.033)	0.111
Q3		0.954 (0.859–1.061)	0.388	1.044 (0.841–1.295)	0.697	0.921 (0.775–1.095)	0.353
Q4		1.040 (0.935–1.157)	0.472	1.105 (0.888–1.375)	0.370	1.044 (0.879–1.240)	0.624
egg-dairy preference pattern							
Q1							
Q2		0.814 (0.734–0.902)	<0.001	0.744 (0.606–0.913)	0.005	0.657 (0.560–0.772)	<0.001
Q3		0.760 (0.685–0.844)	<0.001	0.697 (0.567–0.858)	0.001	0.546 (0.462–0.645)	<0.001
Q4		0.728 (0.656–0.808)	<0.001	0.622 (0.502–0.770)	<0.001	0.465 (0.392–0.553)	<0.001

Models adjusted for gender, age, education level, employment, family history of diseases, exercise, smoking, drinking, sleep, anxiety, depression and other dietary patterns. If the factor was stratified by subgroup, it was not adjusted.

### Association between dietary pattern scores and obesity-related metabolic phenotypes stratified by gender and age

Subgroup analyses were conducted to investigate the associations between dietary patterns and obesity metabolic phenotypes across different gender and age groups. The results are presented in Tables 6 and 7. The analysis demonstrated that the protective associations between the “egg‒dairy preference” pattern and the four phenotypes were particularly strong among females and participants aged 45–60 years. Additionally, the protective association between the “plant preference pattern” and MUNO was more evident among the 45–60 year age group than among those aged 60 years or older. Conversely, the “grain-meat preference pattern” was identified as a risk factor for MHO among individuals aged 60 years or older and females. A protective relationship was observed between the “meat preference pattern” and MUO among individuals aged 45–60 years, whereas an increased risk was noted in those aged 60 years or older.

**Table 6:** Association between quartiles of dietary patterns and metabolic phenotypes of obesity according to gender.

Gender	Dietary patterns	MHNO	MUNO		MHO		MUO	
			OR (95%CI)	*P* value	OR (95%CI)	*P* value	OR (95%CI)	*P* value
Male	Plant preference pattern							
	Q1	-	.	.	.	.	.	.
	Q2		0.864 (0.716–1.043)	0.129	1.115 (0.727–1.709)	0.618	1.043 (0.764–1.424)	0.789
	Q3		0.794 (0.659–0.956)	0.015	0.929 (0.600-1.437)	0.740	1.106 (0.819–1.495)	0.511
	Q4		0.925 (0.774–1.105)	0.391	1.243 (0.828–1.866)	0.295	1.312 (0.983–1.751)	0.065
	Grain-meat preference pattern							
	Q1	-	.	.	.	.	.	.
	Q2		0.886 (0.688–1.140)	0.346	0.955 (0.503–1.814)	0.888	0.935 (0.635–1.376)	0.733
	Q3		0.925 (0.732–1.169)	0.515	1.229 (0.688–2.195)	0.486	0.732 (0.505–1.060)	0.098
	Q4		0.833 (0.666–1.041)	0.108	1.225 (0.702–2.136)	0.475	0.827 (0.586–1.167)	0.279
	Meat preference pattern							
	Q1							
	Q2		0.906 (0.743–1.105)	0.330	0.845 (0.538–1.329)	0.466	0.823 (0.595–1.139)	0.240
	Q3		1.071 (0.884–1.298)	0.481	1.036 (0.679–1.581)	0.869	0.961 (0.707–1.307)	0.799
	Q4		1.179 (0.985–1.412)	0.073	1.029 (0.689–1.537)	0.889	1.152 (0.869–1.526)	0.325
	Egg-dairy preference pattern							
	Q1							
	Q2		0.842 (0.708-1.000)	0.050	0.725 (0.497–1.059)	0.096	0.820 (0.627–1.072)	0.147
	Q3		0.836 (0.699–0.999)	0.049	0.792 (0.541–1.160)	0.232	0.630 (0.470–0.845)	0.002
	Q4		0.880 (0.732–1.059)	0.176	0.571 (0.367–0.891)	0.014	0.932 (0.703–1.237)	0.627
Female	Plant preference pattern							
	Q1	-	.	.	.	.	.	.
	Q2		0.967 (0.854–1.093)	0.589	0.925 (0.722–1.185)	0.536	1.045 (0.854–1.278)	0.671
	Q3		1.019 (0.899–1.155)	0.769	1.150 (0.901–1.469)	0.262	0.942 (0.760–1.167)	0.584
	Q4		0.861 (0.755–0.982)	0.026	1.134 (0.881–1.460)	0.327	0.926 (0.742–1.154)	0.494
	Grain-meat preference pattern							
	Q1	-	.	.	.	.	.	.
	Q2		1.041 (0.928–1.168)	0.491	1.110 (0.879–1.402)	0.381	0.995 (0.818–1.210)	0.960
	Q3		1.025 (0.907–1.157)	0.695	1.054 (0.824–1.347)	0.676	0.877 (0.711–1.082)	0.221
	Q4		1.013 (0.880–1.165)	0.859	1.322 (1.017–1.718)	0.037	1.126 (0.898–1.411)	0.305
	Meat preference pattern							
	Q1	-	.	.	.	.	.	.
	Q2		1.040 (0.919–1.176)	0.536	1.079 (0.844–1.379)	0.543	0.885 (0.719–1.088)	0.245
	Q3		0.913 (0.803–1.037)	0.161	1.051 (0.817–1.352)	0.700	0.929 (0.752–1.149)	0.498
	Q4		0.937 (0.818–1.073)	0.345	1.116 (0.859–1.449)	0.411	0.939 (0.749–1.179)	0.589
	Egg-dairy preference pattern							
	Q1	-	.	.	.	.	.	.
	Q2		0.807 (0.709–0.918)	0.001	0.760 (0.595–0.970)	0.028	0.596 (0.487–0.729)	< 0.001
	Q3		0.747 (0.657–0.850)	< 0.001	0.679 (0.530–0.870)	0.002	0.525 (0.428–0.643)	< 0.001
	Q4		0.696 (0.612–0.791)	< 0.001	0.650 (0.507–0.832)	0.001	0.341 (0.273–0.426)	< 0.001

Models adjusted for age, education level, employment, family history of diseases, exercise, smoking, drinking, sleep, anxiety, depression and other dietary patterns.

**Table 7:** Association between quartiles of dietary patterns and metabolic phenotypes of obesity in different age groups.

Gender	Dietary patterns	MHNO	MUNO		MHO		MUO	
			OR (95%CI)	*P* value	OR (95%CI)	*P* value	OR (95%CI)	*P* value
45–60 years old	Plant preference pattern							
	Q1	-	.	.	.	.	.	.
	Q2		0.923 (0.811–1.049)	0.219	1.052 (0.814–1.360)	0.696	1.077 (0.869–1.337)	0.498
	Q3		0.922 (0.810–1.050)	0.222	1.182 (0.915–1.527)	0.201	1.064 (0.853–1.326)	0.584
	Q4		0.837 (0.731–0.957)	0.009	1.122 (0.859–1.467)	0.398	1.107 (0.887–1.383)	0.368
	Grain-meat preference pattern							
	Q1	-	.	.	.	.	.	.
	Q2		1.097 (0.962–1.251)	0.166	1.031 (0.792–1.341)	0.822	1.047 (0.837–1.309)	0.687
	Q3		1.039 (0.908–1.189)	0.576	1.016 (0.778–1.327)	0.907	0.849 (0.672–1.073)	0.170
	Q4		1.001 (0.867–1.155)	0.993	1.177 (0.893–1.552)	0.247	1.042 (0.824–1.319)	0.729
	Meat preference pattern							
	Q1	-	.	.	.	.	.	.
	Q2		0.957 (0.831–1.103)	0.543	1.015 (0.774–1.332)	0.912	0.725 (0.574–0.916)	0.007
	Q3		0.894 (0.778–1.028)	0.116	0.864 (0.656–1.137)	0.296	0.773 (0.618–0.967)	0.024
	Q4		0.883 (0.769–1.014)	0.079	0.978 (0.749–1.278)	0.870	0.778 (0.625–0.970)	0.026
	Egg-dairy preference pattern							
	Q1							
	Q2		0.788 (0.694–0.896)	< 0.001	0.782 (0.614–0.996)	0.047	0.626 (0.512–0.765)	< 0.001
	Q3		0.690 (0.606–0.785)	< 0.001	0.625 (0.485–0.806)	< 0.001	0.498 (0.403–0.615)	< 0.001
	Q4		0.677 (0.593–0.774)	< 0.001	0.598 (0.459–0.779)	< 0.001	0.448 (0.357–0.561)	< 0.001
≥ 60 years old	Plant preference pattern							
	Q1	-	.	.	.	.	.	.
	Q2		0.962 (0.812–1.141)	0.659	0.806 (0.546–1.190)	0.278	1.008 (0.768–1.324)	0.954
	Q3		0.959 (0.808–1.136)	0.626	0.874 (0.594–1.285)	0.493	0.880 (0.663–1.167)	0.374
	Q4		0.971 (0.820–1.149)	0.728	1.216 (0.850–1.738)	0.284	0.972 (0.739–1.280)	0.841
	Grain-meat preference pattern							
	Q1	-	.	.	.	.	.	.
	Q2		0.912 (0.770–1.080)	0.284	1.210 (0.815–1.795)	0.345	0.940 (0.716–1.236)	0.659
	Q3		1.006 (0.849–1.193)	0.942	1.345 (0.907–1.995)	0.141	0.892 (0.673–1.182)	0.424
	Q4		0.914 (0.765–1.093)	0.326	1.528 (1.023–2.281)	0.038	0.988 (0.741–1.317)	0.934
	Meat preference pattern							
	Q1	-	.	.	.	.	.	.
	Q2		0.979 (0.839–1.142)	0.786	0.915 (0.638–1.312)	0.628	0.983 (0.760–1.271)	0.896
	Q3		0.944 (0.799–1.115)	0.500	1.389 (0.981–1.968)	0.064	1.041 (0.791–1.370)	0.773
	Q4		1.160 (0.975–1.381)	0.094	1.258 (0.851–1.860)	0.250	1.403 (1.061–1.856)	0.018
	Egg-dairy preference pattern							
	Q1	-	.	.	.	.	.	.
	Q2		0.926 (0.778–1.102)	0.387	0.681 (0.463–1.026)	0.051	0.773 (0.590–1.011)	0.060
	Q3		0.954 (0.801–1.137)	0.600	0.900 (0.624–1.297)	0.571	0.708 (0.537–0.932)	0.014
	Q4		0.892 (0.754–1.056)	0.186	0.710 (0.492–1.026)	0.069	0.560 (0.426–0.737)	< 0.001

Models adjusted for sex, education level, employment, family history of diseases, exercise, smoking, drinking, sleep, anxiety, depression and other dietary patterns.

## Discussion

In recent years, a number of studies^34–39^ have revealed the link between chronic diseases and diet in different regions^40–42^ and different characteristic populations^43,44^, which has brought increasing attention to the importance of reasonable healthy eating patterns. Our study found that there are four different dietary patterns among middle-aged and elderly people in three regions of Sichuan Province: the “plant preference” pattern, the “grain-meat preference” pattern, the “meat preference” pattern, and the “egg‒dairy preference” pattern. These patterns reflect the unique dietary characteristics of the regional population.

Previous studies have highlighted the association between dietary patterns and metabolic disorders, indicating that healthier dietary patterns characterized by plant-based foods are linked to better biochemical outcomes and mental health^45^. Owing to their high fiber content and nutrient density, plant-based foods, fruits, and whole grains are known for their beneficial effects on metabolic health. Systematic evaluations and meta-analyses of prospective cohort studies have demonstrated the effects of multiple nutrients contained in plants, such as lowering blood pressure, improving glucose metabolism, and ameliorating dyslipidemia^46^. Therefore, our study uses the “plant preference” pattern as a reference for dietary patterns. These findings suggest that the “meat preference” pattern leads to an increased risk of MUNO compared with the “plant preference” pattern. Some studies^47–51^ have shown that meat intake increases the risk of metabolic syndrome, type 2 diabetes, abdominal obesity, high blood pressure, cardiovascular disease and even several types of cancer. However, the association between meat intake and metabolic diseases remains controversial and requires further investigation^52,53^.

After adjusting for influencing factors, we further examined the multicategorical logistic regression of dietary pattern quartiles with metabolic phenotypes associated with obesity. Our findings revealed that the “plant preference” pattern was beneficial only for MUNO, whereas the “egg-dairy preference” pattern was protective against MUNO, MHO and MUO. The risk decreased with increasing quartile score, indicating that this pattern was superior to the “plant preference” pattern. Research has shown that eggs contain a variety of essential nutrients and bioactive substances, such as high-quality proteins, B vitamins, fat-soluble vitamins, phospholipids and choline^54,55^. A study from Peking University revealed that egg intake was positively associated with several metabolic markers (HDL-C, cholesteryl esters, free cholesterol, phospholipids, etc.) and negatively associated with very-low-density lipoprotein (VLDL) cholesterol in the Chinese population, which may explain the protective effect of moderate egg consumption against cardiovascular diseases^56^. Additionally, milk and dairy intake has been associated with improvements in blood glucose control, blood lipid profiles, and blood pressure regulation, potentially due to the presence of nutrients such as calcium, peptides, and branched-chain amino acids^57–59^.

Our results indicated that the protective association between the “egg-dairy preference” pattern and the four metabolic phenotypes was more evident among females than among males. It is mainly related to the special physiological structure, hormone level and nutritional demand characteristics of women. Women are more prone to calcium loss and osteoporosis due to their physiological characteristics (such as menstruation, pregnancy, lactation, menopause). The egg and milk diet is rich in calcium and vitamin D, which can effectively promote calcium absorption, enhance bone density, and reduce the risk of fracture^60^. In addition, changes in estrogen levels in women (such as a drop in estrogen after menopause) can accelerate bone loss^61^. Calcium and vitamin D in milk and eggs can partially compensate for the negative effects of reduced estrogen.Yao et al.^62^ also showed that higher calcium intake was more significantly positively correlated with lumbar bone mineral density in women than in men.On the other hand, women typically have less muscle mass than men, yet muscles are essential for metabolic health and overall physical function. Numerous studies^63–65^ have shown that high-quality proteins in dairy products, such as whey protein and egg white protein, aid in muscle synthesis and repair, particularly in preventing sarcopenia in middle-aged and older women. The dairy and egg pattern also plays a role in women’s fat metabolism, as proteins and calcium may regulate fat metabolism-related hormones, such as insulin and leptin, thereby improving the distribution of fat in women^65^. For example, in postmenopausal healthy women, daily milk consumption can significantly improve vitamin D status and bone mineral density, as well as have a beneficial effect on blood sugar and lipid levels^66^. Nutrigenomic studies suggest that females and males respond differently to specific diets at the genetic, molecular, and cellular levels^67–69^. Sex differences in response to specific dietary interventions have been reported, particularly those related to n-3 polyunsaturated fatty acid (PUFA) supplementation. The enrichment of a diet with PUFA results in a greater increase in plasma n-3 PUFA in women than in men^70^. This may explain why the “egg-dairy preference” pattern is a favorable dietary pattern for women.

Furthermore, our research revealed that age had an impact on dietary patterns and obesity-related metabolic phenotypes. For example, while the “meat preference” pattern may reduce the risk of MUO in individuals aged 45–60 years, it appears to increase the risk of MUO in those aged 60 years and above. Meat is an important source of high-quality proteins and other nutrients (e.g., protein, vitamins, minerals), On the other hand, meat is relatively high in trans fatty acids and saturated fatty acids, as well as a number of chemicals such as heterocyclic amines, polycyclic aromatic hydrocarbons, nitrates and N-nitroso compounds, which have been shown to induce inflammation and increase the risk of oxidative stress^71–73^.This age-related difference may be attributed to changes in physiological structure, immune function, hormonal levels, and metabolic status associated with aging. As individuals age, organ function declines, and their digestive and absorptive capacities diminish. Eating large amounts of meat may exacerbate metabolic dysfunction in people aged 60 and older, increasing their susceptibility to metabolic disorders.

There are several limitations in this study. First, dietary frequency data were obtained via questionnaire analysis, which inherently introduced recall bias among participants. To address this, we conducted a pilot survey prior to formal data collection, referencing relevant literature and consulting experts to resolve issues identified during the pilot phase. Multistage quality control measures were implemented before, during, and after data collection to minimize potential biases. Despite these efforts, residual recall bias may persist in the survey results. Second, while the study adjusted for many potential confounders in the multiclassification logistic model, unknown confounders may still interfere with the study results. Third, the study sample was recruited exclusively from three regions of Sichuan Province, which exhibit distinct dietary patterns, thus restricting the applicability of the findings to other Chinese populations. This limitation hinders the generalizability of results to other regions of Sichuan or other Chinese provinces. Fourth, this survey revealed that higher adherence to the “grain-meat preference” dietary pattern was associated with the highest prevalence of MHO. However, the failure to differentiate grains into refined (linked to adverse metabolic effects) and whole grains (known for their protective properties) introduced ambiguity into the conclusions, as the nutritional impacts of these grain subtypes may differ substantially. Finally, due to the observational nature of this study, our findings can only reveal a potential association between dietary patterns and four obesity metabolic phenotypes, and the causal relationship cannot be determined yet. Further verification of these results in future longitudinal cohort or interventional trials is essential to address this limitation.

## Conclusion

Overall, this study provides valuable insights into the relationship between dietary patterns and the metabolic phenotype of obesity among middle-aged and elderly individuals. The findings also emphasize the importance of considering gender and age differences when assessing the impact of dietary patterns on metabolic health. The results indicate that the “egg-dairy preference” pattern is more favorable for preventing and controlling MUNO, MHO, and MUO. Additionally, the “plant preference” pattern benefits MUNO, whereas the “grain-meat preference” pattern is detrimental to MHO. These findings underscore the importance of promoting healthier dietary patterns, such as those rich in eggs, dairies, and plant-based foods and low in processed meats, to improve metabolic health and reduce the risk of obesity-related complications among middle-aged and elderly individuals. Further research is warranted to validate these findings and explore potential mechanisms underlying the observed associations.

## Data Availability

The data supporting the findings of this study are available from the corresponding author upon reasonable request.
